# From Conservative Measures to Surgical Interventions, Treatment Approaches for Cubital Tunnel Syndrome: A Comprehensive Review

**DOI:** 10.7759/cureus.51262

**Published:** 2023-12-29

**Authors:** Saurabh Shelke, Ratnakar Ambade, Aditi Shelke

**Affiliations:** 1 Surgery, Jawaharlal Nehru Medical College, Datta Meghe Institute of Higher Education & Research, Wardha, IND; 2 Orthopaedic Surgery, Jawaharlal Nehru Medical College, Datta Meghe Institute of Higher Education & Research, Wardha, IND; 3 Medicine, Jawaharlal Nehru Medical College, Datta Meghe Institute of Higher Education & Research, Wardha, IND

**Keywords:** early intervention, patient-centered care, surgical intervention, nerve compression, treatment approaches, cubital tunnel syndrome

## Abstract

Cubital tunnel syndrome (CuTS) is a neuropathic condition characterized by the compression or irritation of the ulnar nerve at the elbow, resulting in a wide spectrum of symptoms ranging from pain and numbness to muscle weakness and impaired hand function. This comprehensive review delves into the diverse landscape of CuTS treatment approaches, emphasizing the importance of early intervention. The review explores how these strategies aim to alleviate symptoms and enhance patient well-being by beginning with conservative measures encompassing rest, splinting, medications, physical therapy, and lifestyle adjustments. Non-surgical medical interventions, including nerve gliding exercises, ultrasound-guided nerve injections, and orthotic devices, are considered alternative therapies for symptom relief. Surgical interventions, such as decompression procedures and emerging techniques, are discussed in detail, highlighting their indications and expected outcomes. Throughout this review, the critical role of patient-centered care is underscored, emphasizing the need for tailored treatment plans that respect individual preferences and goals. Recognizing the unique nature of each CuTS case, shared decision-making between patients and healthcare providers is advocated, ensuring that interventions align with specific patient needs. As research advances, promising developments in diagnosis, surgical techniques, and drug therapies offer hope for more effective management of CuTS, paving the way for improved symptom relief and enhanced nerve function.

## Introduction and background

Cubital tunnel syndrome (CuTS), also known as ulnar neuropathy at the elbow, is a prevalent and often debilitating condition that affects the ulnar nerve as it traverses the cubital tunnel at the elbow. This condition can result in pain, tingling, weakness, and sensory disturbances in the forearm and hand. Understanding the various treatment approaches for CuTS is essential for healthcare professionals, patients, and researchers [1,2]. The ulnar nerve provides sensory and motor innervation to the forearm and hand, making it a critical component of upper limb function. When the ulnar nerve is compromised within the cubital tunnel, it can lead to a range of symptoms, including numbness and tingling in the ring and small fingers, muscle weakness in the hand, and pain along the inner aspect of the elbow [2].

The significance of comprehending the treatment options for CuTS cannot be overstated. CuTS is a condition that can significantly impact an individual's quality of life, affecting their ability to perform everyday tasks, work, and enjoy recreational activities. Without appropriate intervention, the symptoms of CuTS can worsen over time, leading to functional impairment and permanent nerve damage. Timely and effective treatment is crucial for alleviating pain, preserving nerve function, and improving patients' well-being [3]. Furthermore, CuTS represents a complex medical challenge with various potential approaches to management. Each patient's presentation is unique, and treatment decisions should be tailored to their needs, symptoms, and preferences. A thorough understanding of available treatment modalities is vital for healthcare providers to make informed recommendations and collaborate with patients to develop personalized treatment plans [4]. The primary purpose of this comprehensive review is to explore and evaluate the full spectrum of treatment approaches for CuTS, ranging from conservative measures to surgical interventions.

## Review

Clinical presentation and diagnosis

Signs and Symptoms

Numbness and tingling: A hallmark of CuTS, patients commonly experience numbness and tingling sensations, particularly in the ring and small fingers. These abnormal sensations, known as paresthesia, occur due to pressure or irritation of the ulnar nerve within the cubital tunnel. This sensory disturbance can range from mild to severe and may be intermittent or persistent [1].

Weakness: Muscle weakness in the hand, particularly in grip strength, is another prevalent symptom of CuTS. As the condition progresses, the weakened muscles can affect dexterity and fine motor skills, making tasks like holding objects or manipulating small items more challenging [5].

Pain: Pain is a frequently reported symptom in CuTS and can manifest in various ways. Patients may experience discomfort along the inner aspect of the elbow, radiating pain down the forearm and into the hand, or even localized pain at the site of nerve compression. Pain can vary in intensity and may be exacerbated during activities that place additional pressure on the ulnar nerve [6].

Clumsiness: CuTS-induced muscle weakness and altered sensation can lead to clumsiness in affected individuals. This may result in unintentional dropping of objects or difficulties with tasks that require precise hand movements [7].

Burning sensation: Some CuTS patients describe a burning or pins and needles sensation in the forearm and hand. This burning discomfort can be distressing and particularly noticeable during symptom exacerbation episodes [8].

Sensory loss: CuTS can result in sensory loss, with individuals experiencing a reduced ability to perceive touch, temperature, or pain in the affected fingers, especially the ring and small fingers [9].

Worsening symptoms at night: CuTS symptoms often worsen when the elbow is flexed during sleep. This nocturnal exacerbation is due to increased pressure on the ulnar nerve in a bent position, leading to disturbed sleep patterns and discomfort [2].

Diagnostic Methods

Physical examination: During the physical examination of a patient suspected of having CuTS, several key clinical tests and observations can provide valuable diagnostic information. Tinel's Sign, involving tapping the ulnar nerve at the cubital tunnel, may elicit a tingling sensation or discomfort along the ulnar nerve distribution, confirming nerve irritation or compression at this site. Froment's Sign, a test of thumb strength and adductor pollicis function, can reveal weakness, which is indicative of CuTS due to ulnar nerve dysfunction. The elbow flexion test, where the elbow is flexed for an extended period during the examination, can reproduce symptoms, providing further evidence for diagnosis. Additionally, observing muscle atrophy, particularly in the hypothenar and interosseous muscles, can signify chronic CuTS and its impact on muscle function [10].

Electrodiagnostic studies: Electrodiagnostic studies play a crucial role in confirming and assessing the severity of CuTS. Nerve conduction studies (NCSs) are used to evaluate the speed and amplitude of nerve impulses, with a hallmark finding being slowed nerve conduction across the ulnar nerve at the cubital tunnel. Electromyography (EMG) examines the electrical activity of muscles supplied by the ulnar nerve, helping identify muscle denervation and dysfunction. Needle EMG, a more detailed variant, can pinpoint the precise location and severity of nerve compression, aiding in the assessment and management of CuTS [11].

Imaging: Imaging studies are essential for identifying structural abnormalities and confirming the diagnosis of CuTS. X-rays assess bony abnormalities in the elbow, such as osteophytes or fractures, which can contribute to nerve compression within the cubital tunnel. MRI is particularly valuable for visualizing soft tissue structures, allowing the identification of anatomical variations, space-occupying lesions, and the precise location of nerve compression. Ultrasonography, with its high-resolution capabilities, can provide real-time assessment of nerve morphology and dynamic nerve motion during elbow flexion and extension, facilitating diagnosis and localization of nerve compression. These diagnostic tools, when used in combination, provide a comprehensive evaluation of CuTS, enabling healthcare professionals to formulate an effective treatment plan for their patients [12].

Conservative treatment approaches

Conservative treatment approaches for CuTS are essential initial steps in managing the condition, aiming to alleviate symptoms and improve functional outcomes without surgical intervention. These strategies encompass a range of non-invasive methods and lifestyle adjustments [13]. Activity modification plays a pivotal role in CuTS management, as patients are counseled to avoid or reduce activities that worsen symptoms, such as repetitive elbow flexion or direct pressure on the ulnar nerve. Adjustments in work tasks and hobbies may be necessary to prevent symptom exacerbation. Educating patients on proper elbow positioning, particularly during sleep, alleviates nighttime symptoms [14].

Splinting and bracing are often employed, with elbow splints designed to maintain the elbow in a slightly extended position during sleep, thereby reducing pressure on the ulnar nerve. Customized elbow braces or orthotics may also be beneficial in providing support and preventing excessive flexion or nerve compression during daily activities [15].

Medications, including nonsteroidal anti-inflammatory drugs (NSAIDs) and corticosteroids, may be prescribed to manage pain and inflammation associated with CuTS. NSAIDs offer short-term relief and improve comfort, while corticosteroid injections can reduce swelling and alleviate symptoms, although their effects may be temporary [16].

Physical therapy interventions include stretching and nerve gliding exercises, which aim to enhance ulnar nerve mobility and reduce compression within the cubital tunnel. Strengthening exercises for the muscles around the elbow and hand can also improve stability and reduce strain on the ulnar nerve [17]. Occupational therapy plays a vital role in CuTS management through ergonomic assessments of the patient's workplace, making recommendations to reduce ulnar nerve strain during work-related tasks. Occupational therapists may also design custom splints or braces tailored to a patient's work and daily activities needs [18].

Ergonomic modifications encompass adjustments to the patient's workstation, including proper chair and desk height, keyboard and mouse placement, and forearm support to minimize elbow flexion. Adding cushioning or padding to patients' tools and equipment frequently used can also help reduce direct pressure on the ulnar nerve [19]. Lifestyle changes are encouraged, such as maintaining a healthy body weight to reduce pressure on the ulnar nerve, mainly if excess weight is carried in the upper body. Patients are advised to avoid resting their elbows on hard surfaces for extended periods, which can exacerbate symptoms. Additionally, quitting smoking is recommended, as smoking can impair blood flow and tissue healing, potentially hindering overall recovery from CuTS. Overall, conservative approaches offer a comprehensive and holistic strategy for managing CuTS to improve patient comfort and function while minimizing the need for surgical intervention [20].

Non-surgical medical interventions

Non-surgical medical interventions for CuTS provide a range of options to alleviate symptoms and improve nerve function without surgical procedures. Nerve gliding exercises are integral to physical therapy, focusing on enhancing ulnar nerve mobility and reducing compression. Ulnar nerve flossing, range of motion exercises, and stretching and strengthening routines collectively contribute to relieving pressure on the nerve and promoting its proper functioning [13].

Ultrasound-guided nerve injections, another non-surgical approach, are beneficial in cases of inflammation and pain. By precisely delivering corticosteroid medication to the affected area under ultrasound guidance, inflammation is reduced, and symptoms are alleviated. This technique offers temporary relief and is beneficial during acute exacerbations or when conservative measures alone are insufficient [2].

Orthotics and assistive devices play a crucial role in day-to-day management. Elbow braces and splints maintain the elbow in an extended position, reducing pressure on the ulnar nerve during sleep and activities involving prolonged elbow flexion. Wrist splints maintain the wrist in a neutral position to minimize nerve compression. Ergonomic devices and workplace modifications reduce the risk of exacerbating symptoms during desk work, enhancing overall comfort and function. Additionally, cushioned padding on frequently used tools or equipment protects against direct pressure on the ulnar nerve, improving daily quality of life for individuals with CuTS [21]. These non-surgical interventions offer a multi-faceted approach to CuTS management, aiming to improve symptoms, preserve nerve function, and enhance the patient's overall well-being. The choice of treatment will depend on the severity of the condition, individual patient needs, and the recommendations of a healthcare professional [22].

Surgical indications

Surgical intervention for CuTS is critical when conservative treatments have proven ineffective or when specific clinical indications suggest surgical management is warranted. The decision to proceed with surgery should be made with careful evaluation, considering the patient's circumstances and the severity of their symptoms [23].

A primary reason for opting for surgical intervention is the failure of conservative treatment measures. Surgery becomes the next logical step when approaches such as rest, splinting, physical therapy, and medications do not offer adequate symptom relief. Additionally, suppose CuTS symptoms persist or worsen over time, resulting in significant functional impairment and an inability to carry out daily activities or work-related tasks. In that case, surgical intervention may be the best option to prevent further deterioration [24].

Severe nerve compression is another compelling reason for surgical intervention. When clinical examination or diagnostic tests reveal visible or palpable compression of the ulnar nerve within the cubital tunnel, surgery is necessary to alleviate the mechanical pressure on the nerve. Severe nerve compression can lead to muscle atrophy, significant sensory loss, and motor deficits in the hand and forearm, making surgical decompression essential to prevent permanent nerve damage and functional deficits [25].

Progressive neurological deficits are indicative of the need for surgical intervention as well. Suppose a patient experiences progressive muscle weakness and muscle atrophy in the hand and forearm muscles supplied by the ulnar nerve. In that case, surgery can help halt further deterioration and promote nerve recovery. Worsening sensory loss, especially in the ring and small fingers, may also necessitate surgery to prevent further sensory deficits [26].

Recurrence of CuTS symptoms is another scenario where surgery may be warranted. Some individuals may experience recurring symptoms despite successful initial conservative treatment or prior surgical intervention. In such cases, surgical revision or exploration may be necessary to address the underlying causes of recurrence, such as scar tissue or persistent nerve compression. Additionally, patients who have experienced complications from a previous surgical procedure for CuTS may require additional surgery to correct the issues and achieve better outcomes.

Surgical treatment options

Various surgical treatment options are available when conservative measures have proven ineffective or when specific indications warrant surgical intervention for CuTS. The choice of surgical technique depends on the patient's condition and the surgeon's expertise. Here are some common surgical approaches for CuTS.

Decompression procedures

Simple decompression: In a simple decompression procedure, the surgeon focuses on releasing the structures that exert pressure on the ulnar nerve within the cubital tunnel. This may involve removing or trimming the tunnel's roof, the cubital tunnel retinaculum, or other tissues causing obstruction. Simple decompression is generally considered less invasive than transposition procedures and primarily seeks to relieve nerve compression while preserving the nerve in its natural position [27].

Subcutaneous transposition: Subcutaneous transposition involves repositioning the ulnar nerve from its original location beneath the skin (subcutaneous) to a new location in front of the medial epicondyle of the humerus, a bone of the upper arm. Relocating the nerve in this manner reduces tension on the ulnar nerve during elbow flexion. Surgeons may secure the nerve in its new position using sutures or attach it to nearby tissues, such as the subcutaneous tissue or muscle [28].

Submuscular transposition: In submuscular transposition, the ulnar nerve is moved to a position beneath a muscle group, typically the flexor-pronator muscles in the forearm. This technique protects the ulnar nerve and lowers the risk of recurrent compression during elbow movements. Positioning the nerve under the muscle protects it from external pressures and friction [29].

In situ decompression: In situ decompression, also called simple decompression with anterior transposition, combines elements of both simple decompression and transposition. During this procedure, the surgeon releases the compressive structures while allowing the ulnar nerve to remain in its original location within the cubital tunnel. This can involve the removal of obstructive tissues or adjustments to the tunnel's anatomy to create more space for the nerve, all without the complete repositioning seen in other transposition techniques [30].

Outcomes and Complications of Surgical Interventions

Outcomes: Surgical interventions for CuTS often yield positive outcomes by effectively alleviating symptoms and enhancing nerve function. After surgery, many patients experience substantial pain relief, reduced numbness and tingling sensations, and improved muscle strength. These improvements contribute to an overall enhancement in the quality of life for CuTS patients. By addressing the root cause of nerve compression and providing more space for the ulnar nerve to function properly, surgical procedures aim to restore optimal nerve health and function in the affected arm and hand [2].

Complications: While surgical interventions for CuTS are generally considered safe and effective, they are not without potential complications. Surgical complications may include infection at the surgical site, hematoma (accumulation of blood at the surgical site), scarring, and nerve injury. Nerve injury during surgery can lead to temporary or, in some cases, permanent sensory or motor deficits in the hand and forearm. Preventing these complications requires careful surgical planning, precise technique, and adherence to infection control measures. Surgeons prioritize protecting and preserving the ulnar nerve during surgical procedures to minimize the risk of nerve damage. Postoperative care and monitoring are also essential to promptly identify and address complications, ensuring the best surgical outcomes for CuTS patients. Patients should communicate openly with their healthcare providers to discuss potential risks and benefits before undergoing surgical intervention for CuTS [31].

Timing of Surgery

Timing considerations: The timing of surgical intervention for CuTS is a critical decision that must be carefully evaluated. The choice of when to proceed with surgery depends on several factors, including the severity of symptoms, the presence of progressive neurological deficits, and the patient's overall health. Early surgical intervention may be recommended in some instances, particularly when CuTS symptoms are severe, rapidly worsening, or causing significant impairment in daily life. Conversely, surgery may be deferred in cases where conservative treatments, such as rest, splinting, medications, and physical therapy, have not been exhausted or when the symptoms are mild and manageable without surgical intervention [32].

Patient assessment: The decision regarding the timing of CuTS surgery should be made in consultation with a specialized hand surgeon or orthopedic surgeon with expertise in treating this condition. A thorough patient assessment is essential during this process. The surgeon will carefully evaluate the patient's specific CuTS condition, the progression of symptoms, and individual factors that may influence the timing of surgery. The assessment considers the physical aspects of CuTS and the patient's goals, preferences, and overall health status [33].

Recovery and rehabilitation: After undergoing CuTS surgery, patients typically embark on a postoperative recovery journey that includes rehabilitation. The timing and intensity of rehabilitation vary depending on several factors, including the specific surgical procedure performed and the patient's progress in the postoperative period. Rehabilitation aims to optimize recovery, restore functional abilities, and prevent complications. Physical therapy is often integral to this process, focusing on regaining strength, range of motion, and fine motor skills. The timing of rehabilitation may also be influenced by the surgeon's recommendations and the patient's response to surgery [34].

Postoperative rehabilitation

Following surgical intervention for CuTS, postoperative rehabilitation is vital in optimizing recovery and achieving the best possible outcomes. This phase of treatment involves a combination of physical therapy, patient education, and support to help patients regain strength, function, and comfort in the affected arm and hand.

Physical Therapy

Pain management: Managing postoperative pain is a primary focus of physical therapy. Physical therapists employ various modalities, including ice and heat therapy and electrical stimulation techniques, to alleviate pain and reduce swelling in the surgical area. These measures contribute to patient comfort during the early stages of recovery [35].

Range of motion exercises: Restoration of full joint range of motion is a crucial goal in CuTS rehabilitation. Patients are guided through exercises designed to improve flexibility and prevent joint stiffness in the elbow, wrist, and fingers. Early initiation of these exercises helps maintain joint mobility and prevent contractures [36].

Strengthening exercises: Gradual and progressive strengthening exercises are introduced to rebuild muscle strength in the forearm, hand, and affected fingers. Strengthening is a critical component of CuTS rehabilitation, as it enhances grip strength, fine motor control, and overall hand function. By targeting specific muscle groups, physical therapists aid patients in regaining the strength needed for everyday activities [37].

Nerve gliding exercises: Nerve gliding exercises are incorporated into the rehabilitation program to promote the mobility and health of the ulnar nerve. These exercises aim to prevent nerve adhesions, encourage optimal nerve function, and reduce the risk of nerve compression. By gently mobilizing the ulnar nerve, physical therapists contribute to its healing and recovery [38].

Scar management: Effective scar management is essential to ensure that surgical scars heal well and do not contribute to nerve compression or discomfort. Physical therapists may provide techniques such as scar massage, stretching, and applying scar pads or silicone gels to minimize scar tissue adhesions and optimize the scar's appearance and pliability [39].

Functional activities: As patients rehabilitate, physical therapists encourage performing functional activities that mimic their daily tasks. These activities are tailored to each patient's needs and goals, gradually increasing in complexity. Engaging in functional tasks enhances the patient's ability to return to normal activities and regain independence in daily life [40].

Expected Recovery Timeline

Immediate postoperative period (zero to two weeks): The first two weeks following CuTS surgery are characterized by immediate postoperative care. During this period, patients may experience discomfort, swelling, and limited range of motion in the operated arm. The primary focus is pain management, wound care, and early mobilization. Patients are typically advised to perform gentle range of motion exercises to prevent joint stiffness and maintain flexibility [41].

Two to six weeks: The rehabilitation program becomes more intensive as the initial healing process continues. Physical therapy sessions are crucial during this phase. Patients continue with range of motion exercises and begin nerve gliding exercises to promote ulnar nerve mobility and reduce the risk of nerve adhesions. Strengthening exercises are gradually introduced to rebuild muscle strength in the forearm, hand, and affected fingers. Patients may also start reintroducing light activities into their daily routines as tolerated, under the guidance of their healthcare team [42].

Six to twelve weeks: Around the six to twelve-week mark, many patients experience significant pain relief and range of motion improvements. Strengthening exercises become more challenging and targeted to specific muscle groups. Functional activities, designed to mimic daily tasks, are incorporated into therapy sessions to facilitate the return to normal activities. During this phase, patients may notice a gradual improvement in grip strength and fine motor control [43].

Three to six months: By the three to six-month milestone, a substantial level of recovery is often achieved. Strength and function continue to improve, allowing individuals to resume more demanding activities. Many patients can return to work, hobbies, and recreational activities with a reduced risk of symptom recurrence. The focus remains on ongoing rehabilitation and the maintenance of progress [44].

Long-term recovery (six months plus): Full recovery from CuTS surgery may extend beyond six months, and some patients may continue to experience improvements for up to a year or longer. Patients are encouraged to maintain an ongoing exercise program during this phase to prevent recurrence and sustain optimal nerve health. Regular follow-up appointments with healthcare providers may be scheduled to monitor long-term outcomes and address residual symptoms or concerns [45].

Rehabilitation Challenges

Patient compliance: Patient compliance with the prescribed rehabilitation program can be challenging. Some patients may find it difficult to adhere consistently to their rehabilitation exercises and routines. This lack of adherence can impact their progress and the overall effectiveness of their rehabilitation. To address this challenge, patient education and motivation are essential. Healthcare providers can play a pivotal role in explaining the importance of adherence, setting achievable goals, and providing ongoing support and encouragement to keep patients engaged in their recovery process [46].

Nerve recovery: Nerve healing is slow and gradual, and full nerve recovery can take time. Patients may experience persistent numbness or tingling sensations even after successful surgery. Managing patient expectations during this phase is crucial. Patients should be informed that while surgery can alleviate many symptoms, complete resolution of nerve-related issues may require patience and time. Healthcare providers can offer guidance and reassurance to help patients cope with these lingering symptoms and track their progress [47].

Scar tissue: Surgical scars, while typically small, can sometimes lead to the formation of scar tissue or adhesions, potentially causing discomfort or limited mobility. Effective scar management techniques, such as scar massage, stretching exercises, and using scar pads or silicone gels, can help minimize these issues. Physical therapists can guide scar management to optimize the healing and appearance of surgical scars [48].

Individual variability: Recovery timelines can vary significantly among CuTS patients due to the specific surgical procedure performed, individual health characteristics, and the response to rehabilitation. Recognizing and accommodating this variability is essential for healthcare providers. Rehabilitation plans should be tailored to each patient's unique needs and progress, allowing flexibility in the treatment approach. Regular follow-up assessments and adjustments to the rehabilitation plan may be necessary to ensure that patients are on the right track toward recovery [49].

Alternative and emerging therapies

Platelet-Rich Plasma (PRP) Therapy

PRP therapy is an innovative approach within regenerative medicine, offering a potential avenue for promoting tissue healing and repair. This therapy capitalizes on the body's natural healing mechanisms by utilizing a patient's blood plasma enriched with a higher concentration of platelets than in standard blood. PRP holds significant promise in various medical fields for its ability to accelerate healing processes and reduce inflammation [50].

The mechanism behind PRP therapy hinges on the unique properties of platelets. Platelets are key players in the body's healing cascade, releasing growth factors and bioactive proteins upon activation. These factors, in turn, play a pivotal role in tissue regeneration. In the context of CuTS, PRP therapy is believed to exert its effects by delivering a concentrated dose of growth factors and bioactive proteins to the site of nerve compression within the cubital tunnel. This targeted application is designed to stimulate tissue regeneration, foster nerve healing, and mitigate local inflammation, all crucial aspects of CuTS management [51].

The application of PRP therapy in CuTS treatment typically involves the direct administration of PRP injections into the cubital tunnel region where the ulnar nerve is compressed. The primary objective of this approach is to facilitate nerve recovery and alleviate the various distressing symptoms associated with CuTS, including pain, numbness, and weakness. PRP therapy is considered a minimally invasive option, potentially offering CuTS patients a novel avenue for symptom relief and improved nerve function [52].

Despite the promising aspects of PRP therapy, it is essential to note that the research on its effectiveness for CuTS remains relatively limited. While early findings and anecdotal reports suggest potential benefits, more comprehensive studies are needed to establish the treatment's efficacy, optimal application protocols, and long-term outcomes in CuTS. PRP therapy for CuTS is regarded as an emerging treatment option that warrants further investigation and clinical validation to unlock its full potential in improving the lives of individuals affected by this condition [53].

Stem Cell Therapy

Stem cell therapy represents an innovative and promising approach within regenerative medicine, offering potential avenues for tissue repair and regeneration. This therapy harnesses the unique capabilities of stem cells, which can differentiate into various cell types, including nerve cells. Stem cell therapy is being explored as a potential means to address damaged nerves in CuTS by promoting nerve regeneration and healing [54].

The fundamental mechanism behind stem cell therapy lies in the pluripotent nature of stem cells. These cells possess the capacity to transform into a wide range of specialized cell types, making them particularly intriguing for tissue repair applications. In the context of CuTS, stem cell therapy is investigated to facilitate the repair and regeneration of nerves that have been damaged or compressed within the cubital tunnel. By introducing stem cells into the affected area, researchers aim to harness their regenerative potential to encourage the healing of nerves, reduce compression, and ultimately alleviate the debilitating symptoms of CuTS [55].

Applying stem cell therapy for CuTS may involve injecting stem cells directly into the cubital tunnel region. The objective is to create an environment conducive to nerve healing and regeneration, addressing the underlying cause of nerve compression. This approach holds promise as it leverages the body's innate ability to repair and regenerate tissues [56].

It is important to note that stem cell therapy for CuTS is still in the experimental stages, and more research is needed to determine its safety, efficacy, and optimal application protocols. While early studies and preclinical research have shown promise, further investigations, including clinical trials, are necessary to validate its potential as a viable treatment option for CuTS. Stem cell therapy represents an exciting area of exploration in CuTS management, offering hope for future breakthroughs that may enhance the lives of individuals affected by this condition [57].

Emerging Surgical Techniques

As our understanding of CuTS deepens, surgical techniques for its treatment are continually evolving, aiming to improve patient outcomes and reduce postoperative complications. One of the most notable developments in this regard is the exploration of minimally invasive surgical approaches. Surgeons are investigating procedures such as endoscopy and arthroscopy, which can significantly reduce surgical trauma, scarring, and recovery time. These techniques enhance patient comfort and contribute to quicker rehabilitation [58].

Biological augmentation is another promising avenue in CuTS surgery. Emerging surgical techniques involve utilizing biological materials or grafts to support nerve healing and minimize postoperative complications. These materials serve as scaffolds, promoting nerve regeneration and enhancing the overall success of the surgery [59].

In addition to biological augmentation, innovative nerve wraps and conduits are being developed to protect and support nerve regeneration following surgical decompression. These wraps provide a physical barrier, shielding the nerve from external influences and facilitating recovery [60]. Surgeons are also exploring the concept of nerve transfer in CuTS surgery. This technique redirects healthy nerves to the damaged area to restore function and sensation. Nerve transfer offers a novel approach to address nerve damage and improve patient outcomes [61]. Furthermore, the integration of robotic-assisted surgery into CuTS procedures is gaining momentum. Robotic systems enhance surgical precision and allow for more intricate movements in tight spaces, potentially leading to improved outcomes and reduced risks during surgery [62].

Future directions in CuTS treatment

Advances in Diagnosis

Advanced imaging modalities: Ongoing research in medical imaging may lead to the development and widespread use of more advanced techniques for CuTS diagnosis. High-resolution ultrasound and magnetic resonance neurography are two examples. These imaging modalities offer superior visualization of nerve structures, allowing for a more accurate assessment of nerve pathology and pinpointing the exact location of compression or damage. Enhanced imaging can assist physicians in making precise diagnoses and planning tailored treatment strategies [63].

Biomarkers: Researchers are actively exploring identifying specific biomarkers in blood or nerve tissue that can serve as early indicators of CuTS. These biomarkers could revolutionize diagnosis by providing objective and quantifiable evidence of nerve damage or inflammation. Early detection through biomarkers could enable healthcare professionals to initiate treatment promptly, potentially preventing disease progression and improving patient outcomes [64].

Nerve function testing: Advances in nerve function testing are pivotal in detecting and characterizing CuTS. Electrodiagnostic studies, such as nerve conduction studies and electromyography, are continually improving in sensitivity and specificity. These tests allow for a detailed nerve function assessment, confirming CuTS diagnosis and the differentiation from other conditions that may present with similar symptoms. Accurate diagnostic information from these tests is crucial for guiding treatment decisions [65].

Telemedicine and remote monitoring: Integrating telemedicine and remote monitoring technologies can revolutionize CuTS diagnosis and management. Telemedicine allows for more frequent and accessible follow-up assessments of CuTS patients, facilitating early diagnosis and ongoing treatment monitoring. Patients can engage with healthcare professionals remotely, providing updates on their symptoms and progress. This approach not only enhances patient convenience but also enables timely intervention when symptoms worsen or when adjustments to treatment are needed [66].

Innovations in Surgical Techniques

Minimally invasive procedures: The development and refinement of minimally invasive surgical techniques are revolutionizing CuTS surgery. Endoscopy and arthroscopy are becoming more widely adopted due to their potential to reduce surgical trauma, scarring, and recovery times. With smaller incisions and specialized instruments, surgeons can more precisely access and address the cubital tunnel. This minimally invasive approach improves patient comfort and promotes faster rehabilitation, allowing individuals to return to daily activities sooner [67].

Biomechanical studies: Ongoing research into the elbow joint and the cubital tunnel biomechanics is paving the way for innovative surgical techniques. By understanding the complex interactions between tissues and structures in the cubital tunnel, surgeons can develop procedures that optimize nerve decompression while minimizing the risk of recurrent CuTS. These biomechanical insights enable the customization of surgical approaches tailored to each patient's specific anatomical variations and pathology, resulting in more effective and personalized treatments [68].

Nerve regeneration aids: Exploring advanced materials and scaffolds for nerve repair and regeneration holds great promise for CuTS surgeries. Researchers are investigating the use of biological materials, grafts, and nerve wraps to support nerve healing and minimize postoperative complications. These aids act as scaffolds, guiding and promoting nerve regeneration, which can significantly enhance the overall success of CuTS surgery. Such innovations are precious for severe nerve damage or compression [69].

Robotic-assisted surgery: Continued advancements in robotic-assisted surgical systems are transforming CuTS surgeries by offering greater precision and control. These robotic platforms allow surgeons to perform delicate and intricate maneuvers in tight spaces, enhancing the accuracy of nerve decompression and repair. Robotic-assisted surgery improves outcomes and reduces surgical risks, especially in complex cases where fine motor skills and precision are critical [70].

Potential New Drug Therapies

Neuroprotective drugs: One promising avenue of research involves the development of neuroprotective medications. These drugs aim to prevent nerve damage and promote nerve recovery in CuTS. Neuroprotective drugs can mitigate the condition's progression and enhance long-term outcomes by shielding nerves from further harm and facilitating their repair [71].

Anti-inflammatory agents: Inflammation plays a crucial role in the pathogenesis of CuTS. Novel anti-inflammatory agents targeting specific nerve inflammation pathways are being investigated. These medications could provide additional options for CuTS management by reducing pain and swelling, common symptoms of the condition. These drugs may alleviate symptoms and promote nerve healing by dampening the inflammatory response [72].

Biologics: Biologics, which include growth factors and cytokines, hold promise in CuTS treatment. Researchers are exploring their use to support nerve healing and reduce inflammation associated with CuTS. Growth factors and cytokines can stimulate cell proliferation and tissue repair, potentially accelerating nerve regeneration and functional recovery in CuTS patients [73].

Personalized medicine: Advances in genomics and personalized medicine are changing the landscape of CuTS treatment. By studying the genetic factors that influence CuTS susceptibility and response to treatment, personalized medicine may enable tailored therapies. Identifying specific genetic markers associated with CuTS can guide physicians in selecting the most appropriate drug therapies for individual patients and optimizing treatment outcomes [74].

Regenerative therapies: Research into regenerative therapies, such as tissue engineering and stem cell-based approaches, offers exciting possibilities for CuTS management. These therapies enhance nerve regeneration and functional recovery by providing a supportive environment for damaged nerves. Stem cells can differentiate into nerve cells and promote the regeneration of damaged nerve tissue, potentially restoring lost sensation and muscle function in CuTS patients [75].

## Conclusions

CuTS presents complex symptoms and treatment options. Our comprehensive review has illuminated the spectrum of approaches available to address this condition, from conservative measures to surgical interventions. The significance of early intervention cannot be overstated, as it is the linchpin in preventing symptom progression and preserving nerve function. Early diagnosis and prompt treatment are pivotal for achieving optimal outcomes. Throughout this exploration, the central theme has been patient-centered care. Recognizing that each CuTS experience is unique, it is imperative to tailor treatment plans to patients' needs, preferences, and goals. Shared decision-making between patients and healthcare providers is pivotal, fostering collaboration that empowers patients to engage in their care actively. As research continues to unfold, CuTS patients can anticipate innovative and personalized approaches that hold promise for symptom relief and enhanced nerve function, ensuring a brighter outlook for those affected by this condition.
